# Genetic alterations and molecular mechanisms underlying hereditary intrahepatic cholestasis

**DOI:** 10.3389/fphar.2023.1173542

**Published:** 2023-05-31

**Authors:** Shuying Xie, Shizhang Wei, Xiao Ma, Ruilin Wang, Tingting He, Zhao Zhang, Ju Yang, Jiawei Wang, Lei Chang, Manyi Jing, Haotian Li, Xuelin Zhou, Yanling Zhao

**Affiliations:** ^1^ School of Traditional Chinese Medicine, Southern Medical University, Guangzhou, China; ^2^ Department of Anatomy, Histology and Embryology, School of Basic Medical Sciences, Health Science Center, Peking University, Beijing, China; ^3^ Pharmacy College, Chengdu University of Traditional Chinese Medicine, Chengdu, China; ^4^ Department of Pharmacy, 5th Medical Center of Chinese PLA General Hospital, Beijing, China; ^5^ Department of Pharmacy, Chinese PLA General Hospital, Beijing, China; ^6^ Department of Pharmacology, School of Basic Medical Sciences, Capital Medical University, Beijing, China

**Keywords:** heredity, intrahepatic cholestasis, genetic mutation, molecular function, therapy

## Abstract

Hereditary cholestatic liver disease caused by a class of autosomal gene mutations results in jaundice, which involves the abnormality of the synthesis, secretion, and other disorders of bile acids metabolism. Due to the existence of a variety of gene mutations, the clinical manifestations of children are also diverse. There is no unified standard for diagnosis and single detection method, which seriously hinders the development of clinical treatment. Therefore, the mutated genes of hereditary intrahepatic cholestasis were systematically described in this review.

## 1 Introduction

Abnormalities in the synthesis, secretion, metabolism, and excretion of bile acids can result in cholestasis, a condition also known as cholestatic hepatitis. Cholestasis can be categorized as intrahepatic or extrahepatic depending on its location, with hereditary and acquired forms of intrahepatic cholestasis. Hereditary cholestasis includes Progressive familial cholestasis (PFIC), benign recurrent intrahepatic cholestasis (BRIC) (van Mil et al., 2004), Alagille syndrome (McDaniell et al., 2006), Gilbert syndrome (van Dijk et al., 2015), Dubin-Johnson syndrome (Corpechot et al., 2020), and congenital biliary atresia (Bessho and Bezerra, 2011) in newborns. Acquired forms include intrahepatic cholestasis of pregnancy (ICP) (Williamson and Geenes, 2014), drug-induced cholestasis (Lucena et al., 2020), nonalcoholic fatty liver disease (NAFLD) (Targher et al., 2021), obstructive cholestasis (Strnad et al., 2017), TPN-induced cholestasis (Ghosh et al., 2021), primary biliary cirrhosis (PBC) (Floreani et al., 2015), and primary sclerosing cholangitis (PSC) (Yokoda and Carey, 2019). Extrahepatic cholestasis results from injury or obstruction of the extrahepatic bile duct caused by external injury or malignant cholangiocarcinoma (Gao et al., 2020a). Surgical reconstruction of the bile duct or removal of the obstruction can often relieve congenital biliary atresia. Given the complex pathogenesis of hereditary Intrahepatic Cholestasis (HIHC), this review focused on genetic mutations associated with this form of the condition. The categories and details of hereditary cholestasis are shown in Table 1.

**TABLE 1:** Overviews of Hereditary cholestasis.

Subtype of PFIC	Gene	Pathogenic mechanism	Clinical picture	Therapeutic drug
PFIC1	*ATP8B1*	Excessive amino phosphate esters trapped in extracellular lobules can make cell membranes more susceptible to damage from phospholipids and cholestero (Paulusma et al., 2006)	Itchy skin and jaundice are common symptoms of liver disease, which can also cause developmental delays, hepatosplenomegaly (enlarged liver and spleen), diarrhea, and extrahepatic cystic fibrosis (Demeilliers et al., 2006; van der Mark et al., 2014; Gómez-Mellado et al., 2022). In some cases, patients may experience additional health issues such as hypothyroidism, hearing impairment, pancreatitis, pulmonary fibrosis, and Alzheimer’s disease. Other possible complications include hyperglycemia, osteoporosis, and leukemia (Stapelbroek et al., 2009; Li et al., 2015; Wang et al., 2016; Corpechot et al., 2020)	4-PB, SAHA, Nb-DNJ, UDCA, DXM, PEBD, PIBD,I.E., hyperglycemia, osteoporosis, leukemia (Hasegawa et al., 2014; van der Woerd et al., 2016)
		1. Disruption of the structural protein skeleton of the apical membrane can impair bile acid transport (Verhulst et al., 2010)		
		2. Increased mitochondrial Phosphatidylethanolamine (PE) content can lead to changes in mitochondrial oxidative phosphorylation, as well as alterations in low-density lipoprotein receptor (LDLR) levels that result in decreased cholesterol (Gómez-Mellado et al., 2022)		
		3. Abnormal localization of ASBT can impair bile acid reabsorption, leading to diarrhea (van der Mark et al., 2014)		
		4. Reduced expression of CFTR has been linked to extrahepatic cystic fibrosis (Demeilliers et al., 2006)		
PFIC2	*ABCB11*	BSEP utilizes ATP hydrolysis to promote conformational change, thus achieving transmembrane transport of bile acids. After mutation, BSEP has abnormal conformation and bile acid efflux is impaired (Wang et al., 2003)	The patient had elevated serum levels of bile acid, bilirubin, and AFP, as well as jaundice, hepatosplenomegaly, slow growth, and an increased risk for cirrhosis or even liver cancer (Davit-Spraul et al., 2010)	UDCA, 4-PB (Gonzales et al., 2015), rifampicin (Koukoulioti et al., 2021), pravastatin, fexofenadine (Hayashi and Sugiyama, 2009; Gonzales et al., 2012; Naoi et al., 2014; Gonzales et al., 2015), Ivacaftor (Mareux et al., 2020), Gentamicin (Amzal et al., 2021), Cetuximab (Lin et al., 2013)
		1. Ubiquitination of BSEP leads to its breakdown and can disrupt normal bile acid transport (Wang et al., 2002; Hayashi et al., 2005)		
		2. Mutations in the splicosome can cause abnormal structure of BSEP and trigger its degradation by the endoplasmic reticulum. Different mutations may affect the localization and activity of BSEP in cells, but not necessarily its function (Wang et al., 2002; Hayashi et al., 2005)		
		3. The glycosylation level and tubule targeting function are closely related to the proper functioning of BSEP (Mochizuki et al., 2007)		
PFIC3	*ABCB4*	ABCB4 specifically transports PC from hepatocyte tubules to bile canaliculi, where PC binds to cholesterol vesicles to form mixed micelles that protect hepatocytes from the destructive effect of bile acid, which is susceptible to the toxic effect of cholic acid after gene mutation (Ghonem et al., 2014)	The patient exhibits symptoms including jaundice, pruritus, hepatosplenomegaly, portal hypertension, variceal bleeding, pale stools, intellectual disability, decreased bone density, learning disabilities, and other related conditions (Raquel et al., 2015)	UDCA (Jacquemin et al., 2001), iVx-770, Roscovitine, AAV8-h*ABCB4* (Vauthier et al., 2019; Wei et al., 2021)
		1. Decreased levels of phosphatidylcholine (PC) in the bile duct can damage the stability of bile duct vesicles and micelles, increase the proportion of hydrophobic bile acids, and lead to injury of the tubule membrane due to contact with these acids (Oude Elferink RPJ et al., 2006; Delaunay et al., 2016)		
		2. Cholesterol that is not encapsulated by PC is more likely to aggregate and crystallize, obstruct the small bile duct, and cause damage to the liver structure due to the action of gravity between small molecules (Jacquemin, 2001; Oude Elferink RPJ and Paulusma, 2007; Trauner et al., 2007)		
		3. The absence of an enzyme responsible for PC turnover can lead to liver and bile duct inflammation, including hepatocyte necrosis, bile duct dilatation, hyperplasia, portal vein inflammation, and other related conditions (Jacquemin, 2001; Oude Elferink RPJ and Paulusma, 2007; Trauner et al., 2007)		
		4. Mutations in the MDR3 protein at positions 541F, L556R, and Q855L can lead to its misfolding and subsequent degradation by endoplasmic reticulum-related proteins (Delaunay et al., 2016)		
PFIC4	*TJP2*	*TJP2* mutation causes damage primarily to tight junctions. It acts as a tight junction barrier mutation in epithelial and endothelial cells, which is required for proper assembly of tight junctions. *TJP2* mutation causes damage to bile duct and liver polarized cells (Wei et al., 2020)	Patients present with severe cholestasis and high GGT and are at increased risk for severe liver disease, cirrhosis, or liver cancer (Wei et al., 2020)	
		1. Damage to polarized cells in the bile duct and liver can lead to a decrease in the number of tubule membrane transporters		
		2. Hepatic zoning and bile duct formation were inhibited, leading to hepatic sinusoidal endothelial cell injury (Itoh et al., 2021)		
PFIC5	*NR1H4*/*FXR*	FXR is a nuclear receptor that plays a significant role in regulating bile acid synthesis, metabolism, and excretion. Mutations in FXR can cause bile acid accumulation in the liver and impair normal regulation of bile acid homeostasis, leading to damage of liver cells	The patient presented with hepatobiliary enlargement, severe jaundice, elevated serum GGT levels, hepatocyte balloon-like edema, inflammatory cell infiltration, and partial fibrosis (Chen et al., 2004; Chen et al., 2004; Gomez-Ospina et al., 2016)	TCPOBOP (CAR agonist) (Kim et al., 2019)
		1. Abnormal *FXR* can lead to abnormalities in bile acid synthesis, bile acid transporters, and bile acid recovery function (Lin et al., 2018)		
		2. *FXR* affects lipid metabolism, balance in cholesterol metabolism, glucose metabolism, insulin secretion, drug metabolism, carbohydrate metabolism, and can inhibit smooth muscle inflammation and migration. Additionally, *FXR* can affect male testis and prostate homeostasis (Stayrook et al., 2005; Ma et al., 2006; Byun et al., 2019; Garcia et al., 2019; van Zutphen et al., 2019)		
PFIC6 (#619484)	*SLC51A*	OSTα and OSTβ form heterodimer bile salt transporters, which play an important role in the absorption of bile acids from the gut into hepatocytes for further metabolism and secretion into bile (Ballatori et al., 2013; Kunst et al., 2021)	The patient presented with jaundice and chronic diarrhea. Liver biopsy revealed periportal fibrosis (Gao et al., 2020b)	Ursodiol, cholestyramine (Gao et al., 2020b)
		*SLC51A* gene mutation leads to abnormal bile acid reabsorption (Gao et al., 2020b)		
PFIC7 (#619658)	*USP53*	*USP53* mutation caused hypercholanemia and hearing loss in some patients (Alhebbi et al., 2021)	Serum ALT and AST enzyme levels were increased in patients with PFIC7, and partial fibrosis of hepatocytes was observed in liver tissue sections. Some patients have hearing impairment from childhood (Alhebbi et al., 2021; Bull et al., 2021)	Rifampicin, UDCA (Alhebbi et al., 2021)
PFIC8 (#619662)	*KIF12*	The *KIF12* gene encodes a protein involved in intracellular transport, which is important for maintaining normal liver function. Mutations in KIF12 lead to abnormalities in liver polarized cells and disrupt normal intracellular transport processes in liver cells (Stalke et al., 2022)	The child presented with cholestasis and high gamma-GGT (Ünlüsoy Aksu et al., 2019)	
PFIC9 (# 619849)	*ZFYVE19/ANCHR*	ANCHR retards cell shedding by stabilizing cytoplasmic intercellular Bridges (ICBs), prevents premature loss of intercellular chromosome Bridges and accumulation of DNA damage, and ultimately reduces the formation of dikaryotic cells (Massafra et al., 2017)	The patient showed symptoms of hypercholinemia, characterized by high levels of choline in the blood, as well as severe itching (Mandato et al., 2021; Pepe et al., 2023)	Rifampicin, UDCA, Odevixibat (Pepe et al., 2023)
		When biallelic mutations occur in *ZFYVE19*, it can lead to high serum GGT levels, cholestasis, and either ductal plate malformations (DPM) or congenital liver fibrosis (Luan et al., 2021)		
PFIC10 (#619868)	*MYO5B*	*MYO5B* encodes a protein that is important for intracellular transport, and mutations in this gene lead to disruption of normal intestinal and liver function (Aldrian et al., 2021)	In infancy, patient may present with jaundice, pruritus, and enlargement of the liver and spleen. Laboratory tests often show elevated levels of bilirubin TBA, ALT, and AST, but the level of GGT is typically normal. Some patients may also develop diarrhea due to malabsorption of nutrients (Gonzales et al., 2017; Aldrian et al., 2021)	Lysophosphatidic acid (Kaji et al., 2020; Leng et al., 2020)
		1. *MYO5B* can cause microvillus inclusion body disease and affect the targeting of the tubule membrane of BSEP (Qiu et al., 2017)		
		2. In non-polarized cells, it binds to Rab11a to restore transferrin. In polarized cells, it takes part in the localization of apical membrane proteins and the formation of new cavities. Mutations in this gene can lead to functional abnormalities of the transporter (Engevik et al., 2018)		
		3. Changes in *MYO5B* subcellular localization may promote the malignant development of pheochromocytoma and paraganglioma, causing cytoplasmic metastasis to the cell membrane (Girard et al., 2014; Tomić et al., 2020)		
PFIC11 (#619874)	*SEMA7A*	*SEMA7A* is involved in cell signaling and migration, and mutations in this gene can lead to disrupted hepatocyte polarity, causing cholestasis and other liver-related complications (Qiu et al., 2017)	The patient presented with jaundice and elevated serum ALT, AST, and TBA, but normal ALP,GGT, and bilirubin levels (Pan et al., 2021)	UDCA, GSH (Pan et al., 2021)
		Sema7a^R145W^ homozygous mutation causes intrahepatic cholestasis by reducing hepatic *Bsep* and *Mrp2* expression (Pan et al., 2021)		
PFIC12 (#620010)	*VPS33B*	*VPS33B* is a protein involved in intracellular transport and lysosomal function. Mutations in the *VPS33B* gene can result in abnormal liver cell polarity, leading to bile flow disruption and cholestasis (Fu et al., 2019)	The patient presented with jaundice and pruritus, but gamma-GGT levels were not elevated. The patient also has incomplete features of arthrogryposis, renal dysfunction, and cholestasis syndrome (Qiu et al., 2019)	
		The *VPS33B* mutation results in abnormal liver cell polarity (Hanley et al., 2017; Qiu et al., 2019)		
Alagille syndrome	*JAG1*	*JAG1* and *NOTCH2* jointly regulate the ductal structure involved in bile epithelial cell formation. These genes are responsible for regulating the differentiation of bile duct fibroblasts into bile duct cells	In children, obstruction of bile outflow can result in liver tissue enlargement, severe jaundice, and pruritus, as well as physical abnormalities such as a prominent forehead, large internasal septum, and a systolic murmur in the pulmonary arteries, along with intellectual disability. Additionally, more than half of the affected patients were found to have renal insufficiency and peripheral chorioretinopathy (Kamath et al., 2012; da Palma et al., 2021)	Agonist of TGF-β and *SOX9* (Schaub et al., 2018; Adams et al., 2020)
		Mutations in the *JAG1* or *NOTCH2* genes have been linked to biliary deformities and abnormal bile excretion functions which may result in conditions such as Alagille syndrome, a rare genetic disorder that affects the development of bile ducts, as well as other types of cholestasis characterized by reduced bile flow (Hofmann et al., 2010)		
Gilbert syndrome	*UGT1A1*	*UGT1A1* is an enzyme that catalyzes the glucuronidation of bilirubin, increasing its water solubility and promoting its excretion from bile and urine (Fretzayas et al., 2012)	Gilbert syndrome is characterized by intermittent episodes of unconjugated hyperbilirubinemia in affected patients (Fretzayas et al., 2012)	Phenobarbital (Cozzi et al., 2022)
Crigler-Najjar syndrome1		Abnormal bilirubin efflux (Gilbert et al., 2019; Hsu et al., 2022)	Type I presents with severe jaundice within the first few days of life without hemolysis and is often accompanied by bilirubin encephalopathy. On the other hand, Type II is heterozygous, has a late onset, and usually manifests as a milder illnesss (Tukey and Strassburg, 2000; Gailite et al., 2018)	Phototherapy, phenobarbital (Barateiro et al., 2016; Liaqat et al., 2018; Bai et al., 2021)
			However, abnormal cerebellar development and nerve cell death may be observed in some cases of Type II (Bortolussi et al., 2015; Barateiro et al., 2016)	
Dubin-Johnson syndrome	*ABCC2*/*MRP2*	*ABCC2*/*MRP2*, is a member of the ATP-binding (ABC) family of ATP-dependent transporters that plays an important role in the hepatobiliary excretion of conjugated bilirubin molecules. *ABCC2/MRP2* is essential for the detoxification of bilirubin and its proper functioning is necessary for maintaining healthy liver function (Saito et al., 2013)	The patient presented with severe jaundice and nonhemolytic, conjugated hyperbilirubinemia. Serological test results revealed elevated levels of direct bilirubin, transaminases, and total bilirubin accompanied by increased TBA levels (Togawa et al., 2018)	UDCA (Regev et al., 2002)
		*ABCC2/MRP2* mutations can cause a variety of abnormalities such as meaningless exon expression, base deletion, and abnormal *MRP2* transcription due to premature stop codons. In addition, some mutations may result in the retention of the MRP2 protein within the endoplasmic reticulum, thereby blocking bilirubin efflu (Erlinger et al., 2014)		
Rotor syndrome	*SLCO1B1/3*	When OATP1B1 and OATP1B3 proteins are non-functional or absent, bilirubin glucuronide cannot be effectively cleared from the bloodstream (van de Steeg et al., 2012)	The patient presented with severe jaundice, non-hemolytic conjugated hyperbilirubinemia, and elevated levels of direct bilirubin, transaminases, and total bilirubin, accompanied by increased levels of TBA Additionally, the urine tests showed 2–5 times higher porphyrin excretion in RS patients (Strassburg, 2010; Kimura et al., 2021)	Fluid replacement, nutritional support (van de Steeg et al., 2012)
Biliary atresia	*FOXA2, GPC1, ADD3, PKD1L, EFEMP1/3, STIP1, XPNPEP1, REV1, JAG1, MAN1A2, ARF6, CPLANE, JBTS17*	The cause of biliary atresia is not fully understood and can be caused by a range of genetic and non-genetic factors, including exposure to toxins and pollutants, as well as maternal factors such as smoking during pregnancy or taking certain medications during pregnancy. Multiple gene mutations have also been reported to be associated with biliary atresia (Garcia-Barceló et al., 2010; Lam et al., 2021)	Children with BASM may present with splenomegaly, often accompanied by polysplenia or splenic deficiency, as well as situs inversus and vascular abnormalities. If left untreated, these children may develop cholestatic cirrhosis and liver failure within 2 years (Schwarz et al., 2013; Lakshminarayanan and Davenport, 2016)	Bile acid analogues, drugs that reduce the amount of bile acid in the liver, anti-inflammatory, immunosuppressive agents, anti-fibrotic drugs (Bezerra et al., 2018)
		Jaundice and cholestatic cirrhosis caused by intrahepatic and intrahepatic bile duct obstruction (Mack and Sokol, 2005)		
Neonatal sclerosing cholangitis	*DCDC2*	*DCDC2* protein is found in the cytoplasm and cilia of cholangiocytes. However, in individuals with neonatal sclerosing cholangitis who have mutations in the *DCDC2* gene, the mutant protein accumulates in the cytoplasm and is not present in the cilia, which impair the function of cilia that line the bile duct’s epithelial cells, affecting the normal flow of bile (Girard et al., 2016)	The patient presented with a combination of symptoms including jaundice, hepatosplenomegaly, hyperbilirubinemia, cholestasis, and elevated levels of serum GGT activity (Wei et al., 2023)	Bone marrow transplantation (Girard et al., 2012)
Wilson’s disease	*ATP7B*	Mutations in the *ATP7B* gene can lead to the accumulation of copper in the liver and brain, resulting in pathological conditions (Kwo et al., 2017; Ott et al., 2021)	Patients affected by this condition may exhibit various symptoms such as loss of appetite, hepatosplenomegaly, jaundice, and ascites (Członkowska et al., 2018)	D-penicillamine, trientine, dimercaptosuccinic acid, Zinc salts (Yuan et al., 2021)
ICP	*ATP8B1,ABCB11, ABCB4, ABCC2*, *NR1H4, FGF19, MDR3*	Intrahepatic cholestasis of pregnancy(ICP) patients with certain transporter mutations may experience an increase in serum total bile acid concentration, which can raise the likelihood of premature birth or stillbirth, as well as fetal distress (Henry and Welsh, 2015; Floreani and Gervasi, 2016; Vasavan et al., 2021)	Patients with ICP may experience non-specific rash and pruritus, which can worsen at night and cause insomnia, irritability, and even depression. Besides these symptoms, some patients may also experience abdominal pain, nausea, and vomiting (Puljic et al., 2015)	UDCA (Müllenbach et al., 2005; Van Mil et al., 2007)
BRIC1	*ATP8B1*	A heterozygous mutation of *ATP8B1* can result in the abnormal localization of the tubule membrane (Chen et al., 2021; Bing et al., 2022; Mihara et al., 2022)	Benign recurrent cholestasis is characterized by the gradual development of elevated serum levels of bile acids and bilirubin over several weeks to months (Bing et al., 2022)	4-PB (van der Velden et al., 2010)
BRIC2	*ABCB11*	The I498T (1493 T>C) mutation can lead to the retention of *BSEP* within the endoplasmic reticulum, followed by degradation through the proteasome pathway. This process can result in a reduced number of BSEP proteins present in the apical membrane of hepatocytes (Lam et al., 2006)	The patient presented with episodes of recurrent cholestasis (Henkel et al., 2019)	4-PB (Hayashi et al., 2016), UDCA (Jacquemin et al., 2001), rifampicin (Gonzales et al., 2012; Koukoulioti et al., 2021)
LPAC	*ABCB4*	Heterozygous *MDR3* mutations can result in a reduction of bile duct PC content, making it more vulnerable to hydrophobic bile acid damage (Jacquemin, 2001; Oude Elferink RPJ and Paulusma, 2007; Trauner et al., 2007)	Patients with low phospholipid-associated cholelithiasis (LPAC) may present with symptoms such as biliary colic and acute cholangitis (Dong et al., 2021)	UDCA (Khabou et al., 2019)
	*ABCC2*			
DILI	*ABCB4*	The molecular mechanisms underlying drug-induced liver injury (DILI) are multifactorial and complex, involving a combination of genetic susceptibility factors, direct toxicity from the medication itself, and immune-mediated responses (Chalasani et al., 2021)	The patient presented with symptoms of acute liver disease (Es, 2021)	4-PB (Chen et al., 2022)
	*ABCC2*			
	*HLA alleles*			

### 1.1 Progressive familial intrahepatic cholestasis

PFIC (progressive familial intrahepatic cholestasis) is a rare autosomal recessive disorder that affects liver function due to gene mutations. It typically presents as intrahepatic cholestasis in infancy and can lead to end-stage liver disease or failure by preschool age, with few patients surviving without liver transplantation (Yokoda and Carey, 2019).

Symptoms of PFIC usually appear within the first 1–2 months of life and include jaundice, pruritus, severe malabsorption, diarrhea, rickets, growth retardation, and liver and spleen enlargement. Fat-soluble vitamin absorption disorder can cause other symptoms, such as coagulation dysfunction (Amirneni et al., 2020). Notably, serum gamma-glutamyl transferase (GGT) levels are typically normal during cholestasis in PFIC1 and PFIC2, which is unusual for other cholestatic diseases (Poupon et al., 2000).

The most common types of PFIC (PFIC1/2/3) are caused by mutations in the *ATP8B1*, *ABCB11*, and *ABCB4* genes. Other studies have identified mutations in tight junction proteins *TJP2*(tight junction protein 2) and *NR1H4* as contributing to PFIC4/5, as well as mutations in *MYO5B*.

### 1.2 Progressive familial intrahepatic cholestasis 1

PFIC1, also known as Byler’s disease, is a rare disorder caused by mutations or deletions in the *ATP8B1* gene. This gene encodes a protein called ATPase class I type 8B member 1 (*ATP8B1*), which is primarily found in the apical membrane of several types of cells including those in the intestine, stomach, bladder, hepatocytes, bile ducts, and intestines (Davit-Spraul et al., 2010). Under normal conditions, *ATP8B1* helps maintain the asymmetry of the plasma membrane by moving excess lipids from the outer to the inner side of the cell. This process helps protect the cell from harmful hydrophobic bile salts. However, in the presence of *ATP8B1* gene mutations, excess lipids may remain on the outer side of the cell membrane, making it more vulnerable to damage from phospholipids and cholesterol (Paulusma et al., 2006). *ATP8B1* plays a role in forming polarized epithelial cells, which are necessary for the proper expression of the protein and formation of microvilli. *In vitro* studies using small interfering RNA (siRNA) have shown that inhibiting *ATP8B1* expression can disrupt the structure of the membrane in polarized epithelial cells known as Caco2 cells. This disruption can lead to disorganization of the root tip and impair the function of microvilli (Verhulst et al., 2010). Studies on mice with *ATP8B1* gene mutations have shown that their liver cells have reduced ability to transport hydrophobic bile acids, which can lead to cholestasis. These mice also had increased excretion of cholesterol and phosphatidylserine (PS) in the bile, indicating that the normal structure of the cell membrane is disrupted (Gómez-Mellado et al., 2022). This disruption can lead to a loss of asymmetry in the membrane lipids and affect the function of the canalicular membrane. The findings from the studies on mice with *ATP8B1* gene mutations suggest that deficiencies in *ATP8B1* can weaken the resistance of the canalicular membrane to hydrophobic bile salts. This can lead to abnormal bile salt excretion and cholestasis. Additionally, the abnormal structure of the cell membrane can also disrupt the skeleton of the apical excitatory protein, further impairing liver function (Paulusma et al., 2006). The abnormal structure of the cell membrane in *ATP8B1*-deficient liver cells can also impair the function of microvilli, which are important for proper bile acid transport (Verhulst et al., 2010). Additionally, the ability of *ATP8B1* to transport bile acids and form micelles may be closely related to another protein called *CDC50A* (Paulusma et al., 2008; El Sherrif et al., 2013). *ATP8B1* activity is regulated by its autoinhibitory state, which is controlled by the presence of other proteins such as *CDC50A*/*B*. When ATP8B1 forms a complex with these proteins, the autoinhibitory regions at the N and C termini are released, inducing ATP8B1 to enter a phosphorylated state known as E2P that is ready for substrate binding. Interestingly, the presence of bile acids can further promote the self-inhibitory release of *ATP8B1*. This suggests that the regulation of ATP8B1 activity is highly dependent on the concentration and nature of bile acids in the liver (Cheng et al., 2022).

Besides PFIC1, other disorders have been associated with *ATP8B1* dysfunction. Studies have shown that ATP8B1 deficiency can increase the levels of phosphatidylethanolamine (PE) in liver mitochondria, which in turn stimulates mitochondrial oxidative phosphorylation and leads to increased levels of low-density lipoprotein receptor (LDLR). This may provide a possible explanation for the reduced plasma cholesterol levels observed in PFIC1 disease (Gómez-Mellado et al., 2022). Overall, these findings help explain how ATP8B1 deficiencies can lead to PFIC1 and highlight the importance of this protein for proper liver function (Paulusma et al., 2008; El Sherrif et al., 2013).

Mutations in either *ATP8B1* or *CDC50A* in enterocytes can impair the localization of apical sodium-dependent bile acid transporter (ASBT) and affect bile acid reuptake. This impairment may be a cause of diarrhea in PFIC1 patients. ASBT is responsible for absorbing bile acids from the intestine back into the bloodstream, and its dysfunction can lead to malabsorption of bile acids, which can result in loose stools and diarrhea (van der Mark et al., 2014). Loss of *ATP8B1* function and downregulation of cystic fibrosis transmembrane conductance regulator (CFTR) in biliary cells can lead to impaired bile acid excretion and may contribute to the development of extrahepatic cystic fibrosis in PFIC1 patients (Demeilliers et al., 2006). Researchers have explored the use of compounds such as 4-phenylbutyrate (4-PB), which target CFTR modulators, to alleviate intractable pruritus and improve the quality of life in these patients. However, caution should be taken when using such compounds as they may worsen the underlying cystic fibrosis condition (Hasegawa et al., 2014). Several compounds, including 4-PB, suberoylanilide hydroxamic acid (SAHA), and N-butyl deoxynojirimycin (NB-DNJ), have been found to improve the impaired plasma membrane transport function of *ATP8B1*. These compounds may hold promise as potential treatments for PFIC1 and related conditions. However, further research is needed to determine the safety and efficacy of these compounds in clinical settings. (van der Woerd et al., 2016). *ATP8B1* mutations can lead to organ dysfunction outside of the liver, including hypothyroidism (Li et al., 2015), hearing impairment (Stapelbroek et al., 2009), pancreatitis, and pulmonary fibrosis (Wang et al., 2016). In addition, studies have suggested that *ATP8B1* genetic variants may be related to resilience in Alzheimer’s disease (Corpechot et al., 2020), although further research is needed to confirm this association. These findings highlight the importance of early detection and proper management of *ATP8B1*-related conditions to prevent complications and improve patient outcomes.

Treatment options for patients with PFIC1 include ursodeoxycholic acid (UDCA), which can relieve pruritus and jaundice. Other drugs such as S-adenosyl-methionine have shown promise in treating related conditions like primary biliary cholangitis (PBC), primary sclerosing cholangitis (PSC), and ICP. Glucocorticoids and other immunosuppressants may also be effective, but their use are limited due to potential side effects such as hyperglycemia, osteoporosis, and leukemia. Surgical procedures like partial external bile duct transfer (PEBD), partial internal bile duct shunt (PIBD), and ileal exclusion (I.E.,) have been used to reduce bile acid reabsorption and alleviate symptoms of cholestasis. PEBD has shown some therapeutic effect, with improvements in serum bile acid concentrations, liver function indexes, and slowed progression of liver fibrosis in most PFIC1/2 patients. PIBD transfers bile from the gallbladder to the colon, reducing bile acid reabsorption and lowering serum bile acid and bilirubin levels. I.E., bypasses the distal ileum to reduce bile acid reabsorption. Odevixibat, an oral agent that interrupts the enterohepatic circulation of bile acids, has shown effectiveness in reducing serum bile acid levels and pruritus, and improving quality of life in children with PFIC1/2. However, more research is needed to determine the long-term safety and efficacy of this drug (Thompson et al., 2022). Liver transplantation remains the most effective treatment for PFIC1, but a shortage of liver donors poses a significant challenge. While liver transplantation can provide a cure for PFIC1, it carries the risk of complications and requires lifelong immunosuppressive therapy. Therefore, alternative treatments such as medical therapy and surgical interventions are important options to consider before resorting to liver transplantation. Additionally, efforts to increase awareness about organ donation and improve organ allocation may help address the issue of donor shortages.

Studies have shown that the absence of *ATP8B1* can result in incomplete polarization of human monocyte-derived macrophages (HMDMs) into M2c phenotype, which may aid in identifying PFIC1 patients without significant pathogenic mutations in *ATP8B1*. This suggests that *ATP8B1* plays a role in promoting the differentiation of HMDMs into M2c macrophages. Therefore, analyzing the M2c phenotype of patient HMDMs may be helpful in the diagnosis of PFIC1 and BRIC1. Flow cytometry can be used to detect the M2c phenotype and determine whether there is an *ATP8B1* gene mutation in PFIC1 patients. This approach may provide a non-invasive and reliable diagnostic tool for these rare liver diseases (Mizutani et al., 2021).

### 1.3 Progressive familial cholestasis PFIC2

Both progressive familial intrahepatic cholestasis type 2 (PFIC2) and benign recurrent intrahepatic cholestasis (BRIC2) are caused by mutations in the *ABCB11* gene, which encodes the BSEP protein. Compared to PFIC1, PFIC2 typically has an earlier onset and more rapid progression, often leading to end-stage liver disease or liver failure before adulthood. Patients with PFIC2 may have elevated plasma levels of bile acids, bilirubin, and alpha-fetoprotein (AFP), as well as symptoms such as jaundice, pruritus, liver and spleen enlargement, and slow growth. Liver biopsies of patients with PFIC2 often show varying degrees of inflammation, including dilation of bile ducts, loss of microvilli, structural abnormalities in mitochondria, accumulation of cell granules in the bile duct lumen, fibrosis, and eventually cirrhosis. Children with PFIC2 are also at increased risk of developing liver nodules, which can be dysplastic and may progress to cancer.

BSEP uses ATP hydrolysis to power conformational changes that enable transmembrane transport of substrates, which is necessary for BSEP to transport bile acids. The energy from ATP hydrolysis allows BSEP to change its shape and move substrates across the hepatocyte canalicular membrane and into bile for excretion (Wang et al., 2003). BSEP is expressed primarily in the liver and functions as the main bile salt export pump. Unlike other P-glycoproteins, which transport a variety of compounds, BSEP specifically catalyzes the transport of hydrophobic bile salts such as glycine- and taurine-conjugated bile acids across the canalicular membrane of hepatocytes. In addition to transporting bile acids, BSEP also plays a role in lipid secretion from hepatocytes into bile. This process is important for regulating bile acid homeostasis and preventing accumulation of toxic levels of bile acids in the liver. Impaired BSEP function can disrupt bile acid homeostasis and lead to cholestasis and liver damage, as seen in PFIC2 and BRIC. As the primary bile salt export pump in the liver, BSEP is an important target for developing therapies for cholestatic liver diseases (Hayashi et al., 2005; Hayashi et al., 2012).

The expression of BSEP mRNA and protein can vary widely among individuals and is tightly regulated by transcriptional mechanisms (Ho et al., 2010). Upon activation, farnesoid X receptor (FXR) forms a heterodimer with the retinoid X receptor alpha (RXRα) and binds to specific DNA sequences in the promoter region of the *ABCB11* gene, known as the *FXR* response element (FXRE). This binding event leads to activation of BSEP transcription and increased expression levels of BSEP mRNA and protein. The degree of activation of BSEP transcription by *FXR* is positively correlated with the concentration of CDCA. In other words, higher concentrations of CDCA lead to greater activation of BSEP transcription and increased expression of BSEP (Plass et al., 2002; Baghdasaryan et al., 2014).

Bile acids not only serve as substrates for BSEP transport, but also play a role in regulating BSEP expression and localization. For example, the bile acid taurocholate can promote the targeting of BSEP to the canalicular membrane by activating the exchange protein directly activated by cAMP (EPAC)/liver kinase B1 (LKB1)/AMP-activated protein kinase (AMPK) signaling pathway (Homolya et al., 2014). In addition to bile acids, various signaling molecules and transcription factors can indirectly enhance *BSEP* transcription by activating *FXR*. For instance, liver receptor homolog 1 (LRH-1) and nuclear receptor coactivator 6 (*NCOA6*) are known to enhance BSEP expression through *FXR* activation (Ananthanarayanan et al., 2011). Regulation of BSEP localization and turnover also plays a role in regulating its function. BSEP is found to recycle between the plasma membrane and cytoplasm, with internalization and recycling being regulated by various mechanisms. When BSEP completes its function of transporting bile acids across the canalicular membrane, it can be internalized through clathrinid-mediated endocytosis and degraded by lysosomes. Alternatively, BSEP can be recycled back to the canalicular membrane in a Rab11-dependent manner when it next receives a transport signal. Understanding these mechanisms is important for developing therapies that can modulate BSEP function and improve outcomes in cholestatic liver diseases (Aida et al., 2014; Czuba et al., 2018). Some mutations can result in misfolded BSEP proteins that are recognized by the endoplasmic reticulum-associated degradation (ERAD) pathway. Misfolded BSEP proteins may arise due to various mechanisms, including spliceosome mutations, premature termination of transcription, or errors during protein folding. In the ERAD pathway, the misfolded BSEP protein is targeted for degradation via ubiquitination and subsequent proteasomal degradation in the cytoplasm. This leads to reduced levels of functional BSEP protein on the canalicular membrane and impaired bile flow, which can contribute to the development of PFIC2 (Wang et al., 2002; Hayashi et al., 2005). The expression of BSEP protein can be influenced by various factors, including post-translational modifications such as glycosylation. Basic studies have shown that the transport function of rat BSEP protein is dependent on its N-linked glycosylation, as inhibition of glycosylation can impair the targeting of BSEP to the canalicular membrane or lead to abnormal protein folding and degradation in the endoplasmic reticulum. In addition to glycosylation, mutations in the *ABCB11* gene can also affect the targeting function of BSEP. For example, some mutations have been found to affect the interaction between BSEP and myelin and lymphocyte proteins, which are required for proper targeting of BSEP to the canalicular membrane (Mochizuki et al., 2007).

The protein expression level of BSEP can vary among patients with cholestatic liver diseases due to mutations in the *ABCB11* gene. Different mutations may not necessarily affect the function of BSEP, but can alter its localization and activity within cells, resulting in distinct clinical phenotypes. For example, studies have shown that two mutations in the *ABCB11* gene, p. A570T and p. R1050C, can lead to higher levels of BSEP expression in patients with BRIC2 compared to patients with PFIC2, but lower expression levels compared to patients with ICP (Lam et al., 2007). Ching-Wan Lam and others identified two novel mutations in exons 14 and 18 of the *ABCB11* gene in patients with mild forms of PFIC2. The clinical presentation of these patients was relatively mild compared to classical PFIC2, suggesting a possible genotype-phenotype correlation in *ABCB11* mutations (Lam et al., 2006). In addition, a study by Soroka et al. showed that the localization and turnover of BSEP on the canalicular membrane are regulated by various mechanisms, including vesicle release, anchoring, and internalization pathways. HCLS1-associated protein X-1 (HAX-1), epidermal growth factor receptor pathway substrate 15 (EPS15), adaptor protein-2 (AP-2), and intracellular calcium signaling via the IP3-receptor have been implicated in regulating BSEP trafficking to the canalicular membrane (Soroka and Boyer, 2014).

Some mutations can cause unstable increases in *BSEP* mRNA levels, protein misfolding and instability, loss of transport function, and impaired targeting to the canalicular membrane. To test possible mutated mRNA sites, exonic splicing enhancers (ESE) and exonic splicing silencers (ESdS) can be used. Various computational methods, such as ligand quantitative structure-activity relationship (QSAR), pharmacophore modeling, and nonlinear self-organizing mapping, have been established and validated to analyze high-throughput BSEP *in vitro* drug interaction datasets. While the underlying mechanisms are not yet fully understood, some studies suggest that cholesterol supplements may alleviate the progression of PFIC2. Further research is needed to elucidate the specific mechanisms by which cholesterol affects BSEP function and to determine whether this approach may be a viable therapeutic option for patients with cholestatic liver diseases (Engelmann et al., 2015). Various methods have been used to detect BSEP function and expression, including *Xenopus* oocytes, canalicular membrane vesicles (CMV), BSEP-expressing membrane vesicles, BSEP-expressing cell lines, and sandwich-cultured hepatocytes (SCH) (Cheng et al., 2016). The choice of detection method depends on the specific research question and available resources. Each method has advantages and limitations that must be considered when designing experiments to study BSEP and other hepatic transporters.

The treatment of progressive PFIC2 typically involves medications aimed at improving bile flow and reducing cholestasis. UDCA is considered the first-line therapy for PFIC2, as it can stimulate the transcription of *BSEP* and multidrug resistance-associated protein 2 (MRP2), stabilize liver cell membranes, inhibit hepatocyte apoptosis, and promote bile flow through biliary and hepatic changes. UDCA has demonstrated some therapeutic effect in early-stage PFIC2, but its efficacy is limited in advanced disease where there is severe depletion of BSEP on the canalicular membrane.4 -PB is another medication that has been used to treat severe forms of BSEP deficiency. It can partially correct the tubular localization of mutated BSEP, slow down the ubiquitination of BSEP, and relieve cholestasis in affected individuals. Clinical studies have shown that 4-PB can improve clinical symptoms in some patients with *BSEP* mutations. Other drugs that have shown promise in treating cholestatic liver diseases include pravastatin and fexofenadine (Hayashi and Sugiyama, 2009; Gonzales et al., 2012; Naoi et al., 2014; Gonzales et al., 2015), which have been reported to improve bile excretion and reduce serum bile acid levels in patients with BSEP deficiency. However, further studies are needed to determine the safety and efficacy of these medications in larger patient populations (Hirano et al., 2005; Matsushima et al., 2008). Ivacaftor, a drug used to treat cystic fibrosis, has shown promise as a potential therapy for *BSEP* deficiency. A study by Davit-Spraul et al. demonstrated that ivacaftor can improve BSEP function in patients with PFIC2 who carry the p. Gly308Asp missense mutation (Mareux et al., 2020). In addition, Rachida Amzal and others found that two other compounds, gentamicin and 4-PB, can increase BSEP expression on the canalicular membrane *in vitro*. The study used several cell lines, including HEK293, Can10, HepG2, and MDCK cells, to evaluate the effects of these compounds on BSEP localization and activity. Gentamicin, an antibiotic, was shown to enhance BSEP trafficking to the canalicular membrane by reducing protein degradation, while 4-PB was shown to increase *BSEP* expression through a different mechanism involving transcriptional activation (Amzal et al., 2021).

Liver transplantation is currently the only definitive treatment for PFIC2 with end-stage liver disease. However, PFIC2 can recur after transplantation, particularly in cases where the underlying cause of cholestasis is due to an autoimmune response against BSEP. This condition is known as autoimmune BSEP disease (AIBD), and it is associated with the presence of anti-BSEP antibodies in affected individuals. Detecting the presence of anti-BSEP antibodies can be a useful diagnostic tool for predicting the risk of PFIC2 recurrence after liver transplantation. Immunofluorescence (IF) or immunohistochemistry (IHC) staining of patient liver tissues is commonly used in clinical practice to identify the presence of these antibodies (Kubitz et al., 2015)Anti-BSEP antibody-associated cholestasis severity correlates with the level of anti-BSEP antibodies in the body. Reducing this antibody titer can improve cholestatic hepatitis within days by removing the antibody from plasma using immunosorbent therapy (Kubitz et al., 2016). Cetuximab has also been studied for its potential therapeutic effect on this condition by binding to plasma cells and BSEP antibodies. It is important to note that although reducing the antibody titer may help alleviate symptoms, it may not completely cure the disease. Therefore, ongoing monitoring and management are crucial for effective treatment (Lin et al., 2013).

### 1.4 Progressive familial intrahepatic cholestasis 3

Persistent cholestasis in children caused by *ABCB4* (alias multidrug resistance protein 3 [MDR3]) mutations differs from that of PFIC1/2. The onset of PFIC3 is relatively late, often in adulthood, and presents with symptoms like jaundice, pruritus, hepatosplenomegaly, varicose bleeding caused by portal hypertension, acholic stools, intellectual disability, decreased bone density, and learning disabilities (Raquel et al., 2015). Treatment with UDCA is the first choice for PFIC3, but only 30% of patients respond to it (Jacquemin et al., 2001). Liver transplantation is often viewed as a last resort due to the challenges that come with rejection and postoperative liver degeneration, which can compromise the effectiveness of the procedure. However, other surgical treatments such as complete biliary metastasis, drug transfer of bile acid, and hepatocyte transplantation offer promising new options for treating cholestasis in affected patients (van der Woerd et al., 2017). *ABCB4*/*MDR3* is mainly concentrated in the right lobe of the liver and plays a critical role in the transport of bile acids, phospholipids, and phosphatidylcholine (PC). It acts as a positive regulator of lipid secretion in bile by transporting PC specifically from the bilateral membranes of the tubules to the tubules of hepatocytes. Within the tubules of hepatocytes, PC combines with cholesterol vesicles to form mixed micelles that work synergistically with *ATP8B1* to protect hepatocytes from the damaging effects of bile acids. Overexpression of *MDR3* leads to increased fluorescence intensity of PC, but not other types of phospholipids, during transport from hepatocytes to the bile duct (Ghonem et al., 2014). In individuals with *MDR3* mutations, the level of PC in the bile duct decreases, which disrupts the stability of bile duct vesicles and micelles. This disruption increases the proportion of hydrophobic bile acids and leads to damage caused by contact between these hydrophobic bile acids and the tubule membrane (Oude Elferink RPJ et al., 2006; Delaunay et al., 2016). Cholesterol without PC encapsulation has a greater tendency to aggregate and crystallize due to the gravitational forces between small molecules. This can lead to the obstruction of small bile ducts and damage to the liver structure. The loss of PC turnover enzyme can result in liver and bile duct inflammation, including hepatocyte necrosis, biliary duct expansion, hyperplasia, portal vein inflammation, and other related conditions (Jacquemin, 2001; Oude Elferink RPJ and Paulusma, 2007; Trauner et al., 2007).

The function of *MDR3* is dependent on the specific site of mutation. In general, homozygous mutations lead to PFIC3, while heterozygous mutations result in low phospholipid-associated cholelithiasis (LPAC) (Poupon et al., 2010). Moreover, *MDR3* mutations may also result in PBC, ICP, oral contraceptives-induced cholestasis and drug-induced liver injury (DILI) (Reichert and Lammert, 2018; Vries et al., 2020). Studies have revealed that mutations in specific sites, such as I541F, L556R, and Q855L, can cause misfolding of MDR3 protein, leading to its degradation by endoplasmic reticulum-related proteins. In contrast, mutations at other sites have little effect on MDR3 function (Delaunay et al., 2016). The I54IF mutation in the first nucleotide binding domain (NBD) of MDR3 leads to reduced expression of the protein in the tubule membrane, resulting in its retention in ER/Golgi compartments (Delaunay et al., 2009; Delaunay et al., 2016). In cases of the I541F mutation, MDR3 glycosylation is immature and the protein remains in the cytoplasm. However, at low temperatures, such as 27°C, MDR3 can be expressed on the plasma membrane, promoting its localization within the tubules and restoring normal function to the mutant protein (Delaunay et al., 2009). Some mutation sites in *MDR3*, such as G551D, S1251N, and G1349D, are similar to the gated mutation of *CFTR* or *ABCC7*. The use of *CFTR* enhancers like Ivacaftor (iVx-770) and Roscovitine (Vauthier et al., 2019) can promote normal processing of *MDR3* and increase its expression in the tubule membrane (Delaunay et al., 2017). Recently, gene therapy has shown promising results in restoring phospholipid transport and re-expressing MDR3 protein by targeting liver *hABCB4* mRNA in cells and PFIC3 mouse liver. In mice with PFIC3, liver inflammation, catheter reaction, and fibrosis were significantly reduced following this therapy (Wei et al., 2021). Adeno-associated virus (AAV) is a promising treatment option for PFIC3. After 12 weeks of injecting adeno-associated virus carrying *Abcb4* into PFIC3 mice, all male mice showed a significant curative effect, while 50% of female mice showed improvement. After prolonged treatment, the symptoms of cholestasis in female mice also showed good remission (Weber et al., 2019). Recombinant adeno-associated virus vectors are considered a promising delivery system. The combination of recombinant adeno-associated virus (rAAV) with transposition-mediated somatic cell integration and single-dose injection into *Abcb4* ^*−/−*^ mice at an early age can prevent cholestatic cirrhosis and greatly reduce the incidence of tumors in *Abcb4* ^*−/−*^ mice (Siew et al., 2019). Estrogen and lecithin are also considered significant contributors to PFIC3. Estrogen disturbances can worsen PFIC3 symptoms, especially during pregnancy, leading to severe cholestasis and intractable pruritus. Albumin dialysis or molecular adsorbent recirculation systems (MARS) can alleviate hormone-induced cholestasis exacerbations (Lemoine et al., 2008). Dietary lecithin has been shown to reduce cholestasis symptoms in *Abcb4* ^*−/−*^ mice by decreasing serum levels of cholic acid and bilirubin, and reducing bile duct hyperplasia and activation of fibroblasts around the bile duct (Lamireau et al., 2007). Sem J Aronson et al. found that injecting AAV8-h*ABCB4* into *Abcb4* ^*−/−*^ mice stabilized the expression of ABCB4 protein in their liver, inhibiting the development of PFIC3. J M De Vree et al. discovered that transplantation of liver cells with normal expression of *Mdr3* into *Mdr2* ^*−/−*^ mice could restore phospholipid secretion and improve liver pathological injury (De Vree et al., 2000; Aronson et al., 2019).

### 1.5 Progressive familial intrahepatic cholestasis 4

PFIC4 is a cholestatic disease caused by abnormal function of the TJP2 protein of Tight junction protein ZO-2. *TJP2* plays a significant role in tight junctions and adhesion junctions, acting as a tight junction barrier in epithelial and endothelial cells and being necessary for the proper assembly of tight junctions. Mutations in *TJP2* can also cause hyperkalemia and PFIC1. Patients with *TJP2* mutations typically present with severe cholestasis and high GGT levels. Clinical studies have demonstrated that *TJP2* mutations increase the risk of severe liver disease, cirrhosis, or liver cancer in adolescents and adults (Wei et al., 2020). *ZO-1/2* deficient mice at 6 weeks of age exhibit a higher fatality rate due to damage to the bile duct and liver polarized cells, as well as a decreased number of transporters on the tubule membrane. Liver zonation and bile duct formation are also inhibited, which can lead to injury of hepatic sinusoidal endothelial cells (Itoh et al., 2021). In *Tjp2* knockout mice, levels of Claudin domain-containing protein 1(CLDN1) were reduced, and luciferase isothiocyanate showed impaired tight junctions. Additionally, there were dilated tubules, reduced microvilli density, and decreased expression of the tubule membrane transporter BSEP (Xu et al., 2021). *In vitro* experiments have shown that inhibiting *TJP2* expression using siRNA can inhibit the proliferation of L02 and HepG2 cells, induce cell apoptosis, and cause microfilament disturbance (Tang et al., 2021).

### 1.6 Progressive familial intrahepatic cholestasis 5

PFIC5 is a form of cholestasis that occurs in newborns and is caused by mutations in the nuclear receptor *FXR*/*NR1H4*. This condition can result in liver and gallbladder enlargement, severe jaundice, and elevated serum GGT levels (Chen et al., 2004). *FXR* is a bile acid receptor that binds to bile acids like CDCA, DCA, and DCA, indirectly inhibiting *CYP7A1* and *CYP8B1* while activating transcription factor MafG (MAFG). These factors all play a role in regulating bile acid synthesis, suppressing its production. Inhibition of Hepatic sodium/bile acid cotransporter (*NTCP*) reduces bile acid reabsorption, thereby lowering plasma levels of these compounds. *FXR* also directly recruits histone methyltransferase Histone-arginine methyltransferase CARM1 (*CARM1*) and activates BSEP to promote bile acid secretion. Additionally, inhibition of toll-like receptors can alleviate bile acid-induced cholestatic hepatitis (Lin et al., 2018). In basic studies, *Fxr*/*Shp* double-knockout mice have exhibited a cholestasis phenotype highly similar to human PFIC5. These mice showed severe cholestasis, an increased liver index, and an enlarged gallbladder. Liver pathology revealed increased duct reaction, balloon-like edema of hepatocytes, inflammatory cell infiltration, and partial fibrosis (Gomez-Ospina et al., 2016; Schady and Finegold, 2017). Extracellular vehicles (EVS) have emerged as a potential treatment for cholestatic hepatitis C. McDaniel K et al. found that miRNA Lethal 7, secreted by liver stem cell derived EVS (LSCEVS), can reduce catheter response and biliary fibrosis in mice with cholestasis. *NR1H4* serves as a pivotal mediator of let-7 signal-related inflammation/fibrosis response (McDaniel et al., 2019). In addition to its role in regulating bile acid synthesis and cholestatic hepatitis, *FXR* has also been found to improve the autoimmune function of the central nervous system in an IL10-dependent manner (Hucke et al., 2016). However, *Fx*r/*Shp* knockout mice have exhibited severe cholestasis, an increased liver index, and gallbladder enlargement. Liver pathology analyses of these mice also revealed increased catheter response, hepatocyte balloon-like edema, inflammatory cell infiltration, and partial fibrosis (Kim et al., 2018). The expression of *FXR* can be influenced by various factors, including miRNA-192 (Krattinger et al., 2016) and miRNA-194 (Nie et al., 2017). In addition to its role in regulating bile acid synthesis and cholestatic hepatitis, *FXR* also plays a key role in lipid metabolism (van Zutphen et al., 2019), cholesterol metabolism balance (Byun et al., 2019), glucose metabolism (Ma et al., 2006), insulin secretion, drug metabolism, and carbohydrate metabolism (Stayrook et al., 2005). *FXR* has been found to inhibit smooth muscle inflammation and migration, as well as affect testis and prostate homeostasis (Garcia et al., 2019). Additionally, activation of *FXR* can lead to an extended lifespan through the activation of heterological metabolic pathways (Jiang et al., 2013). *FXR* also plays a crucial role in preventing parenteral nutrition-associated cholestasis (El Kasmi et al., 2022). However, abnormalities in *FXR* function can result in kidney damage. Basic studies have shown that *Fxr* ^−/−^ mice exhibit kidney damage, which may be related to cyc*Nr1h4* (Lu et al., 2020). These studies also found that the activity of the ammonia detoxification enzyme in plasma was reduced, which limited the rate of ammonia decomposition (Massafra et al., 2017). Kang Ho Kim et al. investigated the effects of CAR agonists on cholestatic liver injury in a mouse model of PBC using *Fxr/Shp* double knockout (DKO) mice. They found that treatment with a CAR agonist reduced liver injury and inflammation, as well as decreased the expression of bile acid synthesis genes. The study suggests that CAR agonists may be a potential therapeutic strategy for treating PBC and other cholestatic liver diseases (Kim et al., 2019).

### 1.7 Progressive familial intrahepatic cholestasis 6

PFIC6 (Progressive Familial Intrahepatic Cholestasis type 6) is a rare genetic disorder that affects the liver and digestive system. The disorder is caused by mutations in the *SLC51A* gene which encodes the Organic solute transporter subunit alpha (OSTα) protein. OSTα does form a heterodimeric bile salt transporter with OSTβ, and they play an important role in the uptake of bile acids from the gut into hepatocytes for further metabolism and secretion into bile. It is true that the reduced bile acid reabsorption after *SLC51A* gene mutation leads to decreased bile flow and inhibition of fat digestion, which explains the decreased efficiency of bile acid reabsorption in PFIC6 patients (Ballatori et al., 2013; Kunst et al., 2021). This can result in increased hepatic bile acid synthesis and bile acid accumulation in the liver, leading to cholestatic liver injury and diarrhea (Gao et al., 2020b). However, it should be noted that the diarrhea seen in PFIC6 is mainly due to impaired bile acid transport and not directly related to the reduced efficiency of fat digestion. Additionally, the accumulation of bile acids in the liver can lead to liver damage and cirrhosis if left untreated.

### 1.8 Progressive familial intrahepatic cholestasis 7

PFIC7 (Progressive Familial Intrahepatic Cholestasis type 7) is associated with increased serum levels of the liver enzymes ALT and AST, and partial fibrosis of hepatocytes can be observed in liver tissue sections. Some patients may also develop hearing loss in childhood (Alhebbi et al., 2021; Bull et al., 2021). *USP53* (ubiquitin specific peptidase 53) spliceosome variants can result in the premature termination of the protein, leading to the loss of its functional structure and abnormal metabolic and regulatory interactions. This can contribute to cholestasis in affected individuals (Gezdirici et al., 2023). Jing Zhang et al. did discover that *USP53* mutations may play a role in some of the phenotypic features observed in cases of *TJP2* defect (Zhang et al., 2020). *TJP2* is involved in regulating cell junctions, which are important for maintaining the integrity of tissues such as the liver. Mutations in *TJP2* can cause various liver diseases, including PFIC4. It has been suggested that USP53 may interact with TJP2 and regulate its function in the liver. Additionally, Olga Shatokhina et al. identified biallelic mutations in *USP53* as one of the causes of cholestasis with low γ-GGT (gamma-glutamyltransferase) levels. Low γ-GGT levels are typically seen in patients with low bile flow and can be a sign of intrahepatic cholestasis (Shatokhina et al., 2021).

### 1.9 Progressive familial intrahepatic cholestasis 8

PFIC8 (Progressive Familial Intrahepatic Cholestasis type 8) is caused by mutations in the Kinesin-related protein 12(*KIF12*) gene. The researchers found that these mutations lead to disturbed hepatocyte polarity, which is a disruption in the alignment of cells within the liver tissue. This disruption can impair bile flow and lead to the accumulation of bile acids, causing cholestasis and other liver-related complications. The *KIF12* gene does code for a protein involved in intracellular transport, which is important for maintaining normal liver function. Mutations in *KIF12* can affect the proper functioning of this protein and disrupt the normal intracellular transport processes in hepatocytes (Ünlüsoy Aksu et al., 2019; Stalke et al., 2022).

### 1.11 Progressive familial intrahepatic cholestasis 9

Recent research by Angela Pepe et al. has identified a homozygous mutation in *ANCHR* (Abscission/NoCut checkpoint regulator, *ZFYVE19*) in a 6-year-old girl with chronic cholestasis. The patient exhibited hypercholinemia and severe itching, which were not consistent with classical pathogenic genes associated with PFIC. This newly discovered mutation has been classified as a novel non-syndromic phenotype of PFIC9 (OMIM #619849) (Pepe et al., 2023). *ANCHR* plays a key role in regulating neutron cell shedding during cell division. *ANCHR* delays cell shedding by stabilizing cytokinetic intercellular bridges (ICBs) to prevent premature extinction of intercellular chromosome bridges and accumulation of DNA damage, ultimately reducing the formation of dikaryotic cells (Massafra et al., 2017)^,^ (Bai et al., 2020). *ANCHR* interacts with Charged multivesicular body protein 4c (*CHMP4C*), which helps regulate the timing of abscission and prevents multinucleation in an Aurora-B-dependent manner. Additionally, *ANCHR* controls the late fall-off in DNA separation and midbody reaching through its interaction with BPS4 (Thoresen et al., 2014). Biallelic mutations in *ZFYVE19* can lead to high serum GGT cholestasis and ductal plate malformations (DPM) or congenital liver fibrosis (Mandato et al., 2021). *In vitro* studies have shown that these mutations can cause centrality and axon abnormalities (Luan et al., 2021). Claudia Mandato et al. discovered that a homozygous senseless mutation in *ZFYVE19* resulted in a truncated protein of 222 amino acids, leading to severe cholestasis and jaundice symptoms that manifested 2 months after birth. The patient also had elevated levels of serum bile acids and GGT (Mandato et al., 2021).

### 1.12 Progressive familial intrahepatic cholestasis 10

The researchers found that mutations in a gene called *MYO5B* were responsible for cholestasis and congenital diarrhea. *MYO5B* encodes a protein that is important for intracellular transport, and mutations in this gene lead to disruption of normal intestinal and liver function (Aldrian et al., 2021). In infancy, children with PFIC10 may present with jaundice, pruritus, and enlargement of the liver and spleen. Laboratory tests often show elevated levels of bilirubin, TBA, ALT, and AST, but the level of GGT is typically normal. Some patients may also develop diarrhea due to malabsorption of nutrients (Gonzales et al., 2017; Aldrian et al., 2021). *MYO5B* mutations have been associated with microvillus inclusion body disease (MVID), which affects the targeting of the tubule membrane by BSEP and leads to impaired bile acid excretion, ultimately causing cholestasis (Qiu et al., 2017). The formation of recycling endosomes can also be inhibited by *MYO5B* mutations, which interferes with protein localization between the Golgi apparatus and the cycling endosome (Tzaban et al., 2009; Overeem et al., 2020). This interference can lead to functional expression defects and ultimately result in cholestasis. *MYO5B* binds to Rabgtase, one of the largest families of proteins that regulate membrane transport, to help regulate specific membrane transport pathways. In non-polarized cells, it binds to Rab11a to recover transferrin, while in polarized cells, it plays a role in the localization of apical membrane proteins and the formation of a new lumen (Roland et al., 2011; Schneeberger et al., 2015). Patients with *MYO5B* mutation can exhibit impaired function of the apical circulatory corpuscle pathway in hepatocytes, leading to impaired targeting of BSEP to the tubule membrane (Engevik et al., 2018). This impairment can ultimately result in cholestasis. In addition, damage to the ileum caused by *MYO5B* mutations can lead to increased bile acid reabsorption (Girard et al., 2014). *MYO5B* is also implicated in the malignant development of some pheochromocytomas and paragangliomas, with changes in subcellular localization and metastasis from the cytoplasm to the cell membrane (Tomić et al., 2020). Additionally, lysophosphatidic acid has been shown to improve diarrhea symptoms in mice that are deficient in *MYO5B* (Kaji et al., 2020; Leng et al., 2020).

### 1.13 Progressive familial intrahepatic cholestasis 11

The researchers identified a homozygous R148W mutation in the Semaphorin 7A (*SEMA7A*) gene as a possible cause of PFIC in certain patients. *SEMA7A* is involved in cell signaling and migration, and mutations in this gene can lead to disrupted hepatocyte polarity, causing cholestasis and other liver-related complications. The findings highlight the importance of genetic testing for accurate diagnosis of cholestatic liver disease and the identification of potential treatment targets for these conditions. The study expands our understanding of the genetic basis of PFIC and provides new insights into the molecular mechanisms underlying bile flow disorders (Qiu et al., 2017).

### 1.14 Progressive familial intrahepatic cholestasis 12

PFIC12 (Progressive Familial Intrahepatic Cholestasis type 12) is caused by mutations in the *VPS33B* gene. VPS33B is a protein involved in intracellular transport and lysosomal function. Mutations in the *VPS33B* gene can result in abnormal liver cell polarity, leading to bile flow disruption and cholestasis. Patients with PFIC12 commonly present with additional symptoms beyond cholestasis. The joint curvature and renal dysfunction mentioned are characteristic features of another rare disorder called Arthrogryposis-Renal Dysfunction-Cholestasis (ARC) syndrome, which can be caused by mutations in the same *VPS33B* gene. It should be noted that not all patients with *VPS33B* mutations develop ARC syndrome, but those that do may present with these additional symptoms (Fu et al., 2019).

## 2 Alagille syndrome

Alagille syndrome, also known as neonatal-associated jaundice, is a multi-system autosomal dominant genetic disease (Vandriel et al., 2023).

Obstruction of bile outflow in children can result in enlarged liver tissue, severe jaundice, pruritus, and several physical characteristics such as a prominent forehead, a large internasal septum, and a systolic murmur in the pulmonary arteries. Patients with this condition may also exhibit intellectual disability. More than half of the patients with this condition have been found to have renal insufficiency (Kamath et al., 2012), and peripheral chorioretinopathy can also occur (da Palma et al., 2021).

The majority of patients with this syndrome have mutations in the protein jagged-1 (*JAG1*) gene, while only 1% of patients have mutations in the neurogenic locus notch homolog protein 2 (*NOTCH2*) gene (McDaniell et al., 2006). *JAG1* is a ligand of several Notch receptors and plays an essential role in proper Notch signaling. *JAG1* is also involved in mammalian cardiovascular development, promoting the formation of blood vessels, aortic valves, and pulmonary valves (Andersson et al., 2018). *JAG1* and *NOTCH2* jointly regulate the ductal structure involved in bile epithelial cell formation. These genes are responsible for regulating the differentiation of bile duct fibroblasts into bile duct cells. Mutations in either *JAG1* or *NOTCH2* can lead to biliary deformity and abnormal bile excretion function (Hofmann et al., 2010). Researchers have identified specific targets that can either alleviate or aggravate the course of Alagille disease. For instance, transforming growth factor β^85^ signal and Transcription factor SOX-9 (*SOX9*) (Adams et al., 2020) can promote the formation of the biliary system in hepatocytes and potentially alleviate cholestatic liver disease. On the other hand, THBS2 can inhibit *JAG1*/*NOTCH2* interaction, which may further exacerbate the progression of the disease (Tsai et al., 2016). Partial external bile duct drainage (PEBD) is currently an effective method for treating Alagille syndrome, as it has shown promising results in alleviating symptoms such as jaundice and pruritus. However, PEBD does not cure the underlying issues causing these symptoms and does not address the root cause of the disease. The problem of bile duct excretion in patients with Alagille syndrome has yet to be fully resolved (Olyaei et al., 2001). A Phase 2 clinical study found that maralixibat may have clinical significance for treating Alagille syndrome (Gonzales et al., 2021). Additionally, it has been suggested that enhancing JAGGED/NOTCH signaling through normal random variation, gene therapy, or *NOTCH* agonists may promote intrahepatic duct cell regeneration in patients with Alagille syndrome (Zhao et al., 2022).

## 3 Gilbert syndrome and Crigler-Najjar

Gilbert syndrome is a congenital autosomal recessive disorder characterized by mild unconjugated bilirubin metabolism disorder, which is caused by decreased UDP-glucuronosyltransferase 1A1 (*UGT1A1*) promoter activity (Gilbert et al., 2019; Hsu et al., 2022).

Patients with Gilbert syndrome typically have intermittent episodes of unconjugated hyperbilirubinemia, which means that there is excess bilirubin in the blood that has not been processed by the liver (Fretzayas et al., 2012). However, this is not due to hepatocellular disease (disease affecting the liver cells) or hemolysis (excessive breakdown of red blood cells), but rather a deficiency of the enzyme UDP-glucuronosyltransferase (UGT). This leads to impaired processing of bilirubin and its excretion into the bile, resulting in elevated levels of unconjugated bilirubin in the blood.

It is important to note that *UGT1A1* homozygosity is only a predisposing factor for Gilbert syndrome, as its penetrance is only 50%, most likely due to the influence of modifier genes and the presence of specific haplotypes with other *UGT1A1* genotypes.

*UGT1A1* is an enzyme that catalyzes the glucuronidation of bilirubin, increasing its water solubility and promoting its excretion from bile and urine. *UGT1A1* is primarily found in the liver, small intestine, and colon. Additionally, differential methylation of the −1 CpG site located within the upstream stimulatory factor response element that regulates UGT4A1 expression could explain a portion of the interindividual variability in UGT1A1 in hepatic glucuronidation. Thus, the diagnosis of GS is based on clinical symptoms and laboratory findings, rather than solely on genetic testing (Ehmer et al., 2012; Cooper et al., 2013; Yasar et al., 2013). Clinical studies in patients with Gilbert syndrome have shown that having slightly increased plasma bilirubin concentration or increased bilirubin bioavailability may be beneficial for alleviating metabolic syndrome, cardiovascular disease, and type 2 diabetes (Vitek et al., 2019). This beneficial effect may be due to the inhibition of platelet response and thrombosis by unbound plasma bilirubin. Mutations in *ABCC2* have also been associated with other liver diseases, including LPAC, ICP, and BRIC (Kundur et al., 2015), however, the frequency of these mutations is similar to that in the general population (Corpechot et al., 2020).

Crigler-Najjar syndrome, also known as congenital non-obstructive non-hemolytic jaundice, is a genetic disease caused by autosomal mutations.

It is classified into two types: type I (recessive) and type II (dominant). Type I is homozygous and presents with severe jaundice within 1–4 days of birth without hemolysis. Due to the excellent lipid solubility of unbound bilirubin, it is easy for it to cross the blood-brain barrier, and high concentrations may cause bilirubin encephalopathy (Tukey and Strassburg, 2000). Type II Crigler-Najjar syndrome is a heterozygous form of the disorder that typically presents with mild disease and a late onset. Liver transplantation is an effective treatment for alleviating symptoms of CN1, but it is associated with potential complications, such as postoperative infection, immune rejection, and limited availability of donor organs (Gailite et al., 2018).

Recent basic studies have shown promise in treating CN1 using adeno-associated virus (AAV) injection of *UGT1A1*. While this method has demonstrated success in alleviating the progression of CN1 (Bortolussi et al., 2014; Villiger et al., 2018), there have been reports of brain damage and nucleoomycosis (Yueh et al., 2017) following AAV injection. These adverse effects may be related to the expression of P-glycoprotein, which is enriched in the blood-brain barrier and functions to pump bilirubin out of the brain. Pathological examination of tissues in patients with Crigler-Najjar syndrome has revealed abnormal development of the cerebellum, nerve cell death (Bortolussi et al., 2015), and myelin degeneration (Barateiro et al., 2016). These conditions have been positively associated with bilirubin encephalopathy. Recent research by Kevin A. Strauss et al. has shown that phototherapy can provide protective benefits for patients with CN1 if it is properly used (Strauss et al., 2020). Studies have found that both phototherapy and phenobarbital can effectively lower the levels of unconjugated bilirubin caused by *UGT1A1* gene mutations. These treatments work by increasing the excretion and metabolism of bilirubin from the liver, reducing bilirubin accumulation in the blood and tissues. These findings emphasize the importance of early diagnosis and appropriate management of conditions such as Crigler-Najjar Syndrome Type II to prevent complications and improve patient outcomes (Barateiro et al., 2016; Liaqat et al., 2018; Bai et al., 2021; Cozzi et al., 2022).

## 4 Dubin- Johnson syndrome and rotor syndrome

Dubin-Johnson syndrome (DJs) is an autosomal recessive genetic disorder characterized by non-hemolytic, primarily conjugated hyperbilirubinemia and severe jaundice.

Serological tests typically reveal elevated levels of TBA, direct bilirubin, and glutamine transferase. Histomorphology observations of liver tissue in patients with DJs show dense pigmentation within liver cells. Immunohistochemical analyses of these tissues have revealed decreased levels of MRP2 and increased levels of BSEP (Togawa et al., 2018). Another study has shown that a mutation involving a hydrophobic amino acid, isoleucine, being replaced by phenylalanine results in a 75% reduction of MRP2 tubule membrane localization. This significantly reduces the efficiency of cholic acid efflux (Keitel et al., 2003).

Dubin-Johnson syndrome caused by *ABCC2/MRP2* mutations can be due to various genetic abnormalities, including meaningless exon expression, base deletion, and abnormal transcription leading to premature stop codons. These mutations may lead to bilirubin efflux blockage due to retention of MRP2 protein in the endoplasmic reticulum (Erlinger et al., 2014). *ABCC2*/*MRP2*, is a member of the ATP-binding (ABC) family of ATP-dependent transporters that plays an important role in the hepatobiliary excretion of conjugated bilirubin molecules. *ABCC2/MRP2* is essential for the detoxification of bilirubin and its proper functioning is necessary for maintaining healthy liver function (Saito et al., 2013). The function of ABCC2/MRP2’s nucleotide-binding domain can be affected by missense mutations, such as R768W and Q1382R. The R768W mutation results in abnormal translation and impaired sorting of MRP2 protein, while the Q1382R mutation affects substrate-induced ATP hydrolysis (Hashimoto et al., 2002). Additionally, the abnormal functioning of the PI3Kγ/AKT signaling pathway can lead to decreased utilization of MRP2 membrane surface, potentially impairing its ability to excrete bilirubin and other conjugated waste molecules (Beer et al., 2020). According to Rivka H Regev et al., UDCA was found to have a beneficial therapeutic effect on neonatal Dubin-Johnson syndrome (Regev et al., 2002).

Rotor Syndrome is an autosomal recessive genetic disease characterized by a deficiency in liver uptake and storage of bilirubin (Kimura et al., 2021).

Patients with Rotor Syndrome often present with a range of symptoms, including jaundice, hepatomegaly, and elevated levels of bilirubin and bile acids (Kimura et al., 2021).

Diagnosis of Rotor Syndrome typically involves laboratory tests such as bilirubin and bile acid measurements and imaging studies like MRI or CT scan (Kimura et al., 2021). Management of Rotor Syndrome may include supportive measures such as fluid replacement and nutritional support, as well as medications to reduce bilirubin and bile acid levels. The treatment approach may vary depending on the severity of symptoms and underlying conditions. Rotor Syndrome is thought to result from a deficiency in two transporter proteins, OATP1B1 and OATP1B3, that are responsible for the uptake of bilirubin glucuronide from the blood into the liver. In the absence of functional OATP1B1 and OATP1B3 proteins, bilirubin glucuronide cannot be efficiently cleared from the blood, resulting in hyperbilirubinemia, jaundice, and other symptoms associated with Rotor Syndrome (van de Steeg et al., 2012). Rotor syndrome presents as hyperbilirubinemia with clinical manifestations like those seen in Dubin-Johnson syndrome. However, in patients with rotor syndrome, liver histology was normal and there was no hyperpigmentation in the liver (Kumar et al., 2023). Urine tests in patients with Rotor syndrome have shown increased porphyrin excretion levels ranging from 2 to 5 times higher than the normal range (Strassburg, 2010). It should be noted that certain drugs, such as cyclosporine A, atorvastatin, and rifampicin, can potentially exacerbate symptoms in patients with Rotor Syndrome. These drugs may interfere with the processing and excretion of bilirubin and bile acids in the liver, leading to increased accumulation of these compounds in the blood and tissues. Therefore, caution should be exercised when prescribing medications to patients with Rotor Syndrome, and drug-induced liver injury should be monitored closely. It is important for patients with Rotor Syndrome to inform their healthcare providers about their condition and any medications they are taking, including over-the-counter drugs and herbal supplements (Morais and Machado, 2022).

## 5 Biliary atresia

Biliary atresia, also known as common bile duct dilatation, is a cystic malformation located near the site of common bile duct obstruction (Muise et al., 2006). The prevalence of congenital biliary atresia varies by geography and ethnicity There are significant regional differences in the incidence of BA, ranging from 1:15,000 to 19,000 in Europe and 1:5–10 000 in Japan and China. In addition, both intrahepatic and extrahepatic bile duct obstruction can lead to biliary atresia, but extrahepatic obstruction is typical (Mack and Sokol, 2005; Cavallo et al., 2022).

This condition can be classified into three types based on the location of the atresia. Type I biliary atresia involves no intrahepatic bile duct injury but varying degrees of loss or obstruction in the common bile duct. Choledochojejunostomy is a possible treatment option, and the prognosis for this type is generally good. Type II biliary atresia is associated with intrahepatic bile duct atresia but does not involve damage to the gallbladder or common bile duct. Type III biliary atresia involves hepatic portal atresia (Mieli-Vergani and Vergani, 2009). Biliary atresia can be classified into different types based on its etiology, including Biliary Atresia Splenic Malformation (BASM), cystic atresia, viral biliary atresia, and CMV-IgM+ve associated biliary atresia (Pang et al., 2021). Children with BASM may have malformations of the spleen (polysplenia or absence of spleen), heterotopic viscera (Schwarz et al., 2013), malformations of the heart, and abnormal blood vessels (Lakshminarayanan and Davenport, 2016). These abnormalities can contribute to the development of biliary atresia in affected children. Children with biliary atresia may benefit from Kasai portoenterostomy biliary drainage, which can reduce the need for liver transplantation in 80% of cases. The success of surgery is evaluated based on the level of bilirubin in plasma and the color of the stool (Mieli-Vergani and Vergani, 2009). However, if children still experience persistent cholestasis, cirrhosis combined with liver dysfunction, or portal hypertension after surgery, liver transplantation should be considered. Untreated children may develop cholestatic cirrhosis that progresses to liver failure within 2 years. The etiology of biliary atresia is not completely clear, but it can be caused by genetics, inflammation, viruses, poisons, and other factors (Asai et al., 2015). In addition, liver obstruction or fibrosis due to inflammation may occur in children with diseases such as rotavirus and reovirus (Szavay et al., 2002). Therefore, suppressing the inflammatory response may be a potential treatment option to slow the progression of biliary atresia (Superina et al., 2011).

In the vast majority of patients, currently referred to as isolated biliary atresia, there are still too few reported etiological or pathogenesis clues (Davenport et al., 2021). Isolated biliary atresia is a rare pediatric liver disease in which bile ducts in the liver become inflamed and blocked, leading to liver damage and eventually liver failure (Cui et al., 2013). While the exact cause of isolated biliary atresia is still unknown, research has suggested that it may be the result of both genetic and non-genetic factors (Garcia-Barceló et al., 2010; Lam et al., 2021). Microscopic examination of patients with biliary atresia showed that the cilia of bile duct cells were less in number and shorter in length, and the function of transporting bile acids was downregulated, leading to the accumulation of bile acids, which led to cytotoxicity and ciliary injury (Xia et al., 2006). Genes associated with cilia function such as *MAN1A2, ARF6, CPLANE,* and *JBTS17* mutations can lead to bile duct dysplasia (Chu et al., 2012; So et al., 2020). Other studies have demonstrated that mutations *in FOXA2, GPC1, ADD3, PKD1L1, EFEMP1/3*, *STIP1,* X*PNPEP1, REV1* and *JAG1* genes can increase an individual’s genetic susceptibility to biliary atresia (Cui et al., 2013; Tsai et al., 2014; Tsai et al., 2015; Rajagopalan et al., 2020; Davenport et al., 2021). As for non-genetic mechanisms of pathogenesis, some studies have suggested that viral infections could play a role. Specifically, cytomegalovirus (CMV) has been found in the livers of infants with biliary atresia, leading some researchers to believe that it may trigger an inflammatory response that damages the bile ducts (Bezerra et al., 2014).

Other environmental factors that have been linked to isolated biliary atresia include exposure to toxins and pollutants, as well as maternal factors such as smoking during pregnancy or certain medications taken during pregnancy (Cavallo et al., 2022). In addition, abnormal expression of methylation and microRNA can lead to damage of biliary epithelial cells, which may be related to the pathogenesis of biliary atresia (He et al., 2022). Various treatment options, such as bile acid analogues, drugs that reduce the amount of bile acid in the liver, anti-inflammatory and immunosuppressive agents, and anti-fibrotic drugs, can provide relief from the symptoms of biliary atresia to a certain extent.

## 6 Others

### 6.1 Neonatal sclerosing cholangitis

Neonatal sclerosing cholangitis (NSC) is a severe autosomal recessive liver disease that often results in decompensated biliary cirrhosis during childhood, requiring liver transplantation (Wei et al., 2023).

The patient presented with jaundice, poor growth, and pruritus (Girard et al., 2016).

*DCDC2* gene mutations have been strongly associated with this condition (Wei et al., 2023). *DCDC2* inhibits the classical WNT signaling pathway, regulates ciliary production and length, and is expressed in most organs, including bile duct epithelial cells. Under normal conditions, the DCDC2 protein is found in the cytoplasm and cilia of cholangiocytes. However, in individuals with neonatal sclerosing cholangitis who have mutations in the *DCDC2* gene, the mutant protein accumulates in the cytoplasm and is not present in the cilia (Girard et al., 2016). The *DCDC2* gene is responsible for producing a protein that plays an important role in the development and function of cells in the liver’s bile ducts. When the gene is mutated, this protein may not function properly, leading to inflammation and scarring in the bile ducts. These changes can cause symptoms such as jaundice, poor growth, and itching (Girard et al., 2016). Further research is needed to better understand the mechanisms behind this genetic association and develop effective treatments for this condition (Grammatikopoulos et al., 2016). Georg-Friedrich Vogel et al. found that a variation in the splicing site of the *DCDC2* receptor (c.294–2A>G) can lead to intralobular cholestasis, large catheter sclerosis, and catheter plate deformity (Chen et al., 2018). The patient exhibited symptoms such as jaundice, hepatosplenomegaly, hyperbilirubinemia, cholestasis, and elevated levels of serum GGT activity. A liver biopsy revealed varying degrees of bile duct hyperplasia, portal inflammation, and/or fibrosis (Wei et al., 2023).

### 6.2 Wilson’s disease

Wilson’s disease is an autosomal recessive genetic disorder that presents with loss of appetite, hepatosplenomegaly, jaundice, and ascites (Członkowska et al., 2018).

The abnormal copper metabolism in the body leads to deposition of ceruloplasmin in the liver, which can inhibit bile excretion and cause cholestasis. In severe cases, acute liver failure may occur. If the ceruloplasmin level is low, 24-h urine copper or blood copper tests should be performed. If the diagnosis is not confirmed, molecular genetic analysis of *ATP7B* can also be carried out to confirm the diagnosis (Kwo et al., 2017). In addition to *ABCC2* mutations, other genetic mutations such as those in *MMP9* can also contribute to the development of cholecystitis. *In vitro* studies have shown that *Abcc12* mutation in zebrafish infants can lead to bile duct cell apoptosis. Knocking out *MMP9* results in decreased interlobular bile duct numbers, increased serum AKP and TBIL levels, and aggravated bile duct cell apoptosis (Pham et al., 2021).

Treatment with copper chelators such as D-penicillamine, trientine, dimercaptosuccinic acid and Zinc salts has been shown to significantly improve liver injury and symptoms in patients with Wilson’s disease (Yuan et al., 2021).

### 6.3 Intrahepatic cholestasis of pregnancy

ICP (Walker et al., 2020) is a unique complication that occurs during the second and third trimesters of pregnancy, with incidence rates ranging from 0.2% to 25% (Palmer et al., 2019).

Patients with ICP often experience non-specific rash and pruritus, which can be aggravated at night and lead to insomnia, irritability, and even depression. Abdominal pain, nausea, and vomiting may also occur in some cases. Pruritus usually resolves within 1–2 days after delivery, although in a few patients it can take up to a week. However, the recurrence rate of ICP in subsequent pregnancies is high, at about 60% (Puljic et al., 2015). Laboratory tests have shown that patients with ICP often have increased levels of serum alkaline phosphatase, bile acid, aspartate aminotransferase, and alanine aminotransferase. Due to malabsorption of lipid substances and lipid-soluble vitamins, patients with ICP may experience fatty stool, osteoporosis, hemolysis, and other related conditions. Elevated maternal bile acid levels in ICP can seriously affect the health of newborns and increase the risk of complications such as premature delivery, intrauterine asphyxia, meconium staining of amniotic fluid, and fetal bradycardia (Floreani and Gervasi, 2016). In addition to affecting maternal health, ICP can also have adverse effects on infant cardiac function. Infants born to mothers with untreated ICP may experience abnormal cardiac rhythms, and the severity of the condition is often proportional to maternal bile acid levels (Henry and Welsh, 2015; Vasavan et al., 2021). ICP has been reported to be strongly associated with hepatitis C virus (HCV) infection. HCV can impair the function of ABC protein transporters, leading to disruptions in bile acid transport and metabolism, which may contribute to the development of ICP in affected individuals (Reyes et al., 2000; Hinoshita et al., 2001; Iwata et al., 2011).

Several bile acid-related synthesis and transport proteins are involved in the development of ICP, including *ATP8B1*, *ABCB11*, *ABCB4, ABCC2, NR1H4, FGF19,* and *MDR3*. These proteins play important roles in regulating the metabolism and transport of bile acids in the liver, and mutations or functional abnormalities in these genes may contribute to the pathogenesis of ICP (Kawakita et al., 2015). In addition, 17α-estradiol can induce pregnancy-induced cholestasis by trans-inhibiting the tubulomembrane targeting activity of BSEP. The metabolites of 17α-estradiol are then exported from MRP2 to the tubule lumen. Although BSEP has high homology with *MDR1* and MRP2, these two transporters cannot compensate for cholestasis caused by BSEP mutations because of their low affinity for human primary bile acids. *MDR3* mutations are also closely associated with ICP, and approximately 16% of ICP cases are caused by mutations in this gene (Piechota and Jelski, 2020). *FXR* mutations have also been identified as potential contributors to the development of cholestasis, including ICP. In Caucasian patients with cholestasis, *FXR* mutations have been detected by sequencing. These mutations may be related to the FXR-BSEP pathway, which plays an important role in regulating bile acid homeostasis and transport in the liver. Further studies are needed to understand the precise mechanisms underlying the relationship between *FXR* mutations and ICP (Müllenbach et al., 2005; Van Mil et al., 2007). UDCA is the first-line treatment for ICP as it can reduce pruritus and jaundice in affected patients. However, its effectiveness varies among individuals. UDCA works by increasing the water solubility of bile acids, which reduces their harmful effects on hepatocytes and bile duct epithelial cells. It also protects the cell membrane of hepatocytes and decreases the likelihood of bile acids passing through the placenta to the fetus (Beuers et al., 2015). Although UDCA is often prescribed to reduce symptoms in patients with ICP, a study conducted by British scholars found that it may not effectively prevent adverse perinatal outcomes, as alanine aminotransferase and ICP were not strongly associated with an increased risk of preterm birth or stillbirth (Chappell et al., 2019). In cases where premature delivery is a concern, glucocorticoids may be recommended to reduce the risk of fetal respiratory insufficiency and intraventricular hemorrhage. These medications are typically administered before 34 weeks of gestation to optimize their effectiveness. An individual patient data (IPD) meta-analysis has found that the risk of stillbirth significantly increases when the serum bile acid concentration in patients with ICP reaches 100 μM/L or higher. This highlights the importance of timely diagnosis and treatment of ICP to prevent adverse outcomes for both the mother and the fetus (Ovadia et al., 2019). It is worth mentioning that Peter H Dixon et al. conducted a study to identify genetic mutations associated with ICP. They found that 11 genes were potentially linked to the development of the condition, including *GCKR*, *ABCG5/8*, *ABCB11*, *SCARB2*, *ABCB1/4*, *CYP7A1*, *SERPNA1*, *ENPP7*, *TMEM147*, *SULT2A1*, and *HNF4A33*. Further research is needed to better understand the role these genes play in the pathogenesis of ICP (Dixon et al., 2017; Liu et al., 2020).

### 6.4 Citrin deficiency

Citrin deficiency is a genetic disorder caused by mutations in the *SLC25A13* gene, which encodes an electrogenic aspartate/glutamate antiporter. The condition is inherited in an autosomal recessive pattern and can manifest in different stages. The first stage of Citrin deficiency is characterized by intrahepatic cholestasis in newborns. This represents an early-onset manifestation of the disease and is often associated with jaundice, hepatomegaly, and other related symptoms. Further stages of Citrin deficiency may develop later in life and can include neurological symptoms and other complications (Hayasaka, 2021).

### 6.5 Drug-induced liver injury

DILI is an infrequent but significant adverse drug reaction that can lead to acute liver failure, making it a crucial cause of this condition (Chalasani et al., 2021; Fernandez-Checa et al., 2021).

In cases where DILI is caused by rifampin, treatment with 4-PB has been shown to have a protective effect. This protection is thought to arise from 4-PB inhibiting endoplasmic reticulum (ER) stress and preventing the ubiquitination of MRP2 in hepatocytes. By blocking these processes, 4-PB can help prevent the development of rifampin-induced hepatocyte injury and mitigate the risk of complications associated with DILI (Chen et al., 2022). Numerous genome-wide association studies (GWAS) focusing on DILI have identified various HLA alleles that exhibit a strong association with DILI, albeit being specific to certain drugs. For instance, HLA-B57:01 is linked to DILI caused by flucloxacillin reactions, while HLA-A02:01 and HLA-DRB115:01 are associated with amoxicillin-clavulanic acid (AC)-induced DILI. Additionally, HLA-B35:02 is correlated with minocycline-induced DILI, whereas HLA-A*33:01 is related to terbinafine-induced DILI. These HLA alleles may also potentially play a role in the development of DILI due to other drugs (Cirulli et al., 2019).

Elizabeth T Cirulli et al. conducted a study and found that multiple DILI and cholestasis may be associated with *PTPN22* gene mutations. The *PTPN22* gene encodes a protein involved in regulating immune cell function, which suggests that immune-mediated mechanisms may play a role in the pathogenesis of drug-induced liver injury and cholestasis.

### 6.6 Low-phospholipid-associated cholelithiasis syndrome

LPAC is a rare disease that causes adult intrahepatic cholelithiasis, and it typically affects individuals under 40 years old. Clinical manifestations include biliary colic and acute cholangitis. More than half of LPAC patients have been found to have *ABCB4* mutation, according to Catherine Dong et al. (2021). If left untreated, LPAC syndrome may progress to cholangiocarcinoma (Khabou et al., 2019).

### 6.7 Benign recurrent intrahepatic cholestasis

BRIC is a rare genetic liver disorder that causes intermittent episodes of cholestasis, which is a condition where bile flow from the liver to the small intestine is blocked or impaired.

BRIC is caused by mutations in either the *ATP8B1* gene (type 1) or the *ABCB11* gene (type 2), both of which are involved in the transport of bile acids. The main difference between type 1 and type 2 BRIC is the underlying genetic mutation. Type 1 BRIC is caused by mutations in the *ATP8B1* gene, which regulates bile salt export from the liver. Type 2 BRIC is caused by mutations in the *ABCB11* gene, which encodes a protein called the BSEP that also plays a role in bile acid transport. Both types of BRIC can present with similar symptoms of intermittent attacks of cholestasis, including abdominal pain, jaundice, itching, and fatigue. However, there can be variations in the severity and frequency of these attacks depending on the specific gene mutation involved.

BRIC1 is associated with heterozygous mutations in *ATP8B1*, such as p. T888K (Bing et al., 2022), c.1429 + 2T>G (Mihara et al., 2022), c.1817T>C, and p. I606T (Chen et al., 2021). These mutations are thought to affect the tubular membrane localization of ATP8B1, leading to impaired bile acid transport and cholestasis. Unlike PFIC1, BRIC1/ICP is a milder form of the disease that can recur intermittently and is often responsive to medical therap*y* (Folmer et al., 2009). What’s worse, R Mullenbach et al. found that mutations at two heterozygous sites of *ATP8B1* may lead to ICP, which shows the increasing phosphodiester signal and decreasing phosphomonoester/phosphodiester ratio (Müllenbach et al., 2005).

In contrast, BRIC is a milder form of the disease that typically presents with recurrent episodes of cholestasis and pruritus, but liver damage is generally mild. Diagnosis of both PFIC2 and BRIC typically involves genetic testing, and treatment options depend on the individual patient’s symptoms and disease severity (Henkel et al., 2019). Patients with BRIC can often be managed effectively with medical therapy, such as medications like UDCA and rifampicin, which Improve bile flow and reduce toxicity of bile acids (Koukoulioti et al., 2021). Other medications and interventions, such as bile acid sequestrants, may also be considered depending on the individual patient’s needs. Surgical interventions, such as liver transplantation or biliary diversion surgery, may be necessary for patients with PFIC2 who do not respond to medical therapy.

## 7 Treatments

Children may develop liver fibrosis and even cirrhosis (Cui et al., 2013; Tsai et al., 2014; Tsai et al., 2015). The symptoms of HIHC usually appear during infancy or early childhood and may include jaundice, itching, pale stools, failure to thrive, and growth retardation (Liu et al., 2018; Schonfeld and Brown, 2019). Some forms of HIHC can also lead to progressive liver disease, cirrhosis, and liver failure. Treatment for HIHC generally involves supportive care, such as medication to manage, including UDCA, rifampin and 4-PB (van der Velden et al., 2010; Beuers et al., 2014). In severe cases, liver transplantation may be necessary. In some cases, 4-PB may be used to treat HIHC by increasing the expression of the affected transporter proteins on the canalicular membrane, improving bile flow, and reducing cholestasis-related complications (Naoi et al., 2014; van der Woerd et al., 2016). However, more research is needed to understand the efficacy of 4-PB treatment for HIHC. Mutations in transport proteins such as ATP8B1, BSEP, and MRP2 can result in abnormal positioning of functional proteins, consequently disrupting the normal transportation of bile acids. However, administering 4-PB can partially alleviate the symptoms caused by these proteins, enhance bile acid transport, and reduce the probability of complications relating to cholestasis (Hayashi and Sugiyama, 2007; van der Velden et al., 2010; Hasegawa et al., 2014; Gonzales et al., 2015; Amzal et al., 2021). Additionally, 4-PB is capable of regulating MRP2 ubiquitination by restraining ER stress. By enhancing the efflux of hepatotoxic drugs from the liver, this effect shields against DILI (Chen et al., 2022).

## 8 Conclusion

**FIGURE 1:**
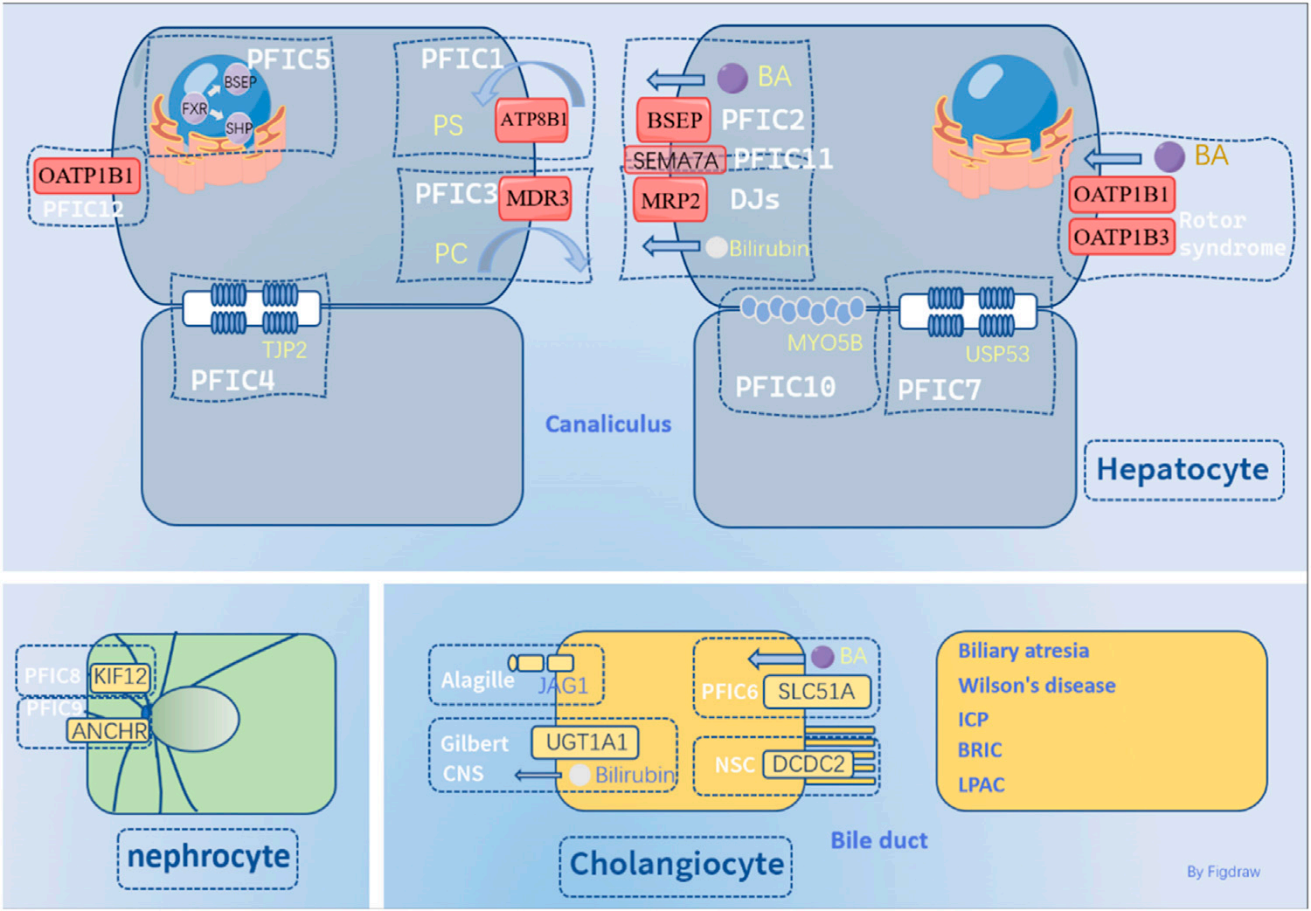
Molecular mechanism behind hereditary intrahepatic cholestasis. PFIC, progressive familial cholestasis; PS, Phosphatidylserine; PC, Phosphatidylcholine; BA, bile acid; DJs, Dubin-Johnson syndrome; ICP, Intrahepatic cholestasis of pregnancy; BRIC, Benign recurrent intrahepatic cholestasis; LPAC, Low phospholipid-associated cholelithiasis. This figure was technically supported by Figdraw.

HIHC is a rare genetic disorder that affects the liver’s ability to secrete bile. It is caused by mutations in several different genes, including *ATP8B1, ABCB11*, *ABCB4* and so on (Wang et al., 2003; Paulusma et al., 2006; Ghonem et al., 2014)*.* These genes play important roles in the transport of bile acids and phospholipids from the hepatocytes into the bile canaliculi, and mutations in these genes can disrupt this process. *NR1H4* mutation leads to abnormal bile acid synthesis rate (Hassan et al., 2022). *SLC51A* mutation results in reduced bile acid reabsorption efficiency (Ballatori et al., 2013; Kunst et al., 2021). Mutations in tight junction proteins (*TJP2, USP53*), *KIF12, MYO5B*, *SEMA7A*, and *VPS33B* lead to impaired hepatocyte polarization, affecting the number of tubular membrane transporters and reducing bile acid transport efficiency (Qiu et al., 2017; Fu et al., 2019; Ünlüsoy Aksu et al., 2019; Wei et al., 2020; Zhang et al., 2020; Aldrian et al., 2021; Stalke et al., 2022). *JAG1* or *NOTCH2* mutations are linked with biliary malformations and abnormal bile excretion (Hofmann et al., 2010). *UGT1A1* and *ABCC2, SLCO1B1/3* mutations result in abnormal bilirubin excretion and metabolism (Hashimoto et al., 2002; van de Steeg et al., 2012; Saito et al., 2013; Gilbert et al., 2019; Hsu et al., 2022). *DCDC2* mutations are associated with cilia and cause restricted bile acid flow (Wei et al., 2023). *ATP7B* mutation leads to copper deposition, causing substantial liver damage that disrupts hepatocyte function (Kwo et al., 2017). Other genes including *FOXA2, GPC1, ADD3, PKD1L, EFEMP1/3, STIP1* were found to be associated with biliary atresia. As shown in Figure 1, the molecular mechanisms behind hereditary intrahepatic cholestasis were summarized.

## Figures and Tables

**Table:** 

**PFIC**	Progressive familial cholestasis
**BRIC**	Benign recurrent intrahepatic cholestasis
**ICP**	Intrahepatic cholestasis pregnancy
**NAFLD**	Nonalcoholic fatty liver disease
**PBC**	Primary biliary cirrhosis
**PSC**	Primary sclerosing cholangitis
**ESLD**	End-stage liver disease
**GGT**	Gamma-glutamyltransferase
**TJP2**	Tight junction protein 2
**FXR**	Farnesoid X receptor
**ATP8B1**	ATPase class I type 8B member 1
**PE**	Phosphatidylethanolamine
**LDLR**	Levels of low-density lipoprotein receptor
**ASBT**	Apical sodium-dependent bile acid transporter
**CFTR**	Cystic fibrosis transmembrane conductance regulator
**4-PB**	4-phenylbutyrate
**SAHA**	suberoylanilide hydroxamic acid
**NB-DNJ**	N-butyl deoxynojirimycin
**PS**	Phosphatidylserine
**CDC42**	Control protein 42 homologous
**CDC50A**	Cell cycle control protein 50A
**UDCA**	Ursodeoxycholic acid
**PEBD**	Partial external bile duct transfer
**PIBD**	Partial internal bile duct shunt
**IE**	Ileal rejection
**HMDMs**	Human monocyte-derived macrophages
**AFP**	Alpha-fetoprotein
**MDR1**	Multidrug transporter P-glycoprotein
**RXRα**	Retinoid X receptor alpha
**FXRE**	*FXR* response element
**LKB1**	liver kinase B1
**AMPK**	AMP-activated protein kinase
**LRH-1**	Liver receptor homolog
**NCOA6**	nuclear receptor coactivator 6
**ERAD**	Endoplasmic reticulum degradation-associated structure
**HAX-1**	HCLS1-associated protein X-1
**EPS15**	Epidermal growth factor receptor pathway substrate 15
**AP-2**	Adaptor protein-2
**ESE**	Exonic splicing enhancers
**ESS**	Exonic splicing silencer
**QSAR**	Quantitative Structure-Activity Relationship
**AIBD**	Autoimmune BSEP disease
**IF**	Immunofluorescence
**IHC**	Immunohistochemistry
**MDR3**	Multidrug resistance protein 3
**LPAC**	Low phospholipid-associated cholelithiasis
**DILI**	Drug-induced liver injury
**CMV**	Canalicular membrane vesicles
**AIBD**	Autoimmune BSEP disease
**PC**	Phosphatidylcholine
**NBD**	Nucleotide binding domain
**AAV**	Adeno-associated virus
**rAAV**	Recombinant adeno-associated virus
**MARS**	Molecular adsorbent recirculation system
**CLDN1**	Claudin domain-containing protein 1
**CARM1**	Histone methyltransferase Histone-arginine methyltransferase CARM1
**OSTα**	Organic solute transporter subunit alpha
**USP53**	Ubiquitin specific peptidase 53
**KIF12**	Kinesin-related protein 12
**ANCHR**	Abscission/NoCut checkpoint regulator
**ICBs**	Intercellular bridges
**CHMP4C**	Charged multivesicular body protein 4c
**DPM**	Ductal plate malformations
**EVS**	Extracellular vehicles
**LSCEVS**	liver stem cell-derived EVS
**MYO5B**	myosin VB
**MVID**	Microvillus inclusion body disease
***SEMA7A***	Semaphorin 7A
**ARC**	Arthrogryposis-Renal Dysfunction-Cholestasis
***JAG1***	Protein jagged-1
***NOTCH2***	Neurogenic locus notch homolog protein 2
**PEBD**	Partial external bile duct drainage
***UGT1A1***	UDP-glucuronosyltransferase 1A1
**DJs**	Dubin -Johnson syndrome
**NSC**	Neonatal sclerosing cholangitis
**BASM**	Biliary Atresia Splenic Malformation
**HCV**	Hepatitis C virus
**IPD**	Individual patient data
**GWAS**	Genome-wide association studies
**PTPN22**	Tyrosine-protein phosphatase non-receptor type 22
**HIHC**	Hereditary Intrahepatic Cholestasis
